# QTL-seq identifies cooked grain elongation QTLs near *soluble starch synthase* and *starch branching enzymes* in rice (*Oryza sativa* L.)

**DOI:** 10.1038/s41598-019-44856-2

**Published:** 2019-06-06

**Authors:** Siwaret Arikit, Samart Wanchana, Srisawat Khanthong, Chatree Saensuk, Tripop Thianthavon, Apichart Vanavichit, Theerayut Toojinda

**Affiliations:** 1 Department of Agronomy, Faculty of Agriculture at Kamphaeng Saen, Kasetsart University Kamphaeng Saen Campus, Nakhon Pathom, 73140 Thailand; 2 Rice Science Center, Kasetsart University Kamphaeng Saen Campus, Nakhon Pathom, 73140 Thailand; 3 National Center for Genetic Engineering and Biotechnology (BIOTEC), National Science and Technology Development Agency (NSTDA), Khlong Luang, Pathum Thani, 12120 Thailand; 4 Faculty of Agriculture at Kamphaeng Saen, Kasetsart University Kamphaeng Saen Campus, Nakhon Pathom, 73140 Thailand

**Keywords:** Natural variation in plants, Plant breeding

## Abstract

Grain quality is one of the main targets that rice breeders focus on to improve elite rice varieties. Several characteristics are considered when determine rice grain quality, such as aroma, amylose content (AC), gelatinization temperature (GT) and, especially, lengthwise grain elongation (GE). GE is a desirable feature in premium rice of high quality, such as India and Pakistan’ Basmati. Inheritance of GE in rice has not been clearly elucidated due to its complex and inconsistent pattern. In this study, we identified QTLs for GE in rice using bulk-segregant analysis (BSA) and whole-genome sequencing based on an F_2_ population segregated for GE as well as AC and GT. We identified two QTLs on chromosome 6, *qGE6.1* and *qGE6.2*, and another QTL on chromosome 4, *qGE4.1*. *qGE6.1* and *qGE6.2* were located near *starch synthase IIa* (*SSIIa)* and *starch branching enzyme III (SBEIII)*, respectively, and *qGE4.1* was located near *starch branching enzyme IIa* (*SBEIIa*). *qGE6.1* was considered to be the major QTL for GE based on this population, and *SSIIa* was suggested to be the best candidate gene associated with the GE trait. The results of this study may be useful for breeding rice with increased grain elongation and different starch properties.

## Introduction

Grain quality is one of the main targets that rice breeders focus on to improve elite rice varieties. Rice grain quality is a complex trait, involving several components such as grain appearance, milling quality, cooking, eating and nutritional quality^1^. In particular, cooking and eating quality traits, such as amylose content (AC), gelatinization temperature (GT), gel consistency and pasting viscosity, and aroma, are key elements in determining the quality of cooked rice^2^. In addition, the rice cooking characteristics were also affected by attributes like water absorption, volume expansion and grain elongation^3^. Rice kernels absorb water and increase their volume by increasing length or width during cooking^4^. The increase in length without significant increase in width or linear elongation is desirable in high-quality premium rice such as Basmati. Grain elongation (GE) is a physical phenomenon that is influenced by the gelatinization temperature^5^. Pre-soaked rice GE is probably linked with low-gelatinization temperature and intermediate amylose^6^.

The inheritance pattern of GE in rice is still difficult to conclude because it is not consistent in different crosses, and very little information is available on GE inheritance patterns in rice. It is challenging to fix the GE character to the desired standard, reflecting the complex inheritance mode^7^. Several studies reported QTLs for GE performed across different genetic backgrounds, including the three QTLs on chromosome 2, 6 and 11^2^; a QTL on chromosome 3^8^; a QTL on chromosome 8^9^ and four QTLs on chromosome 3, 6, 7, and 8^10^. In addition, three QTLs on chromosome 2, 4, and 12 were also identified in non-Basmati varieties^11^. Despite these mapping efforts, only limited information is available on the possible genes and genetic control of GE.

Bulked-segregant analysis (BSA) is an effective method for identifying DNA markers closely linked to the causal gene for a particular phenotype^12,13^. BSA can be applied to any population with significant phenotypic difference^14^. Progeny with extreme phenotypes are used to generate two bulks of DNA samples, instead of the entire population, and DNA markers with differences between the two bulks are screened. A method called “QTL-seq” has recently been developed for QTL identification, combining BSA and whole-genome re-sequencing of two DNA bulks of progeny (each with 20–50 individuals) with extreme phenotypic values^15^. QTL-seq facilitates the rapid identification of QTLs as it does not require the development and genotyping of DNA markers, the most time-consuming and expensive procedures required for the conventional QTL analysis. QTL-seq has been widely used to detect QTLs for a number of traits in several crops, such as blast disease and seedling vigor in rice^15^, cold tolerance in *Oryza rufipogon*^16^, early flowering in cucumber^17^, fruit weight and locule number in tomato^18^, 100-seed weight and root/total plant dry weight ratio in chickpea^19^, and rust and late leaf spot resistance in groundnut^20^.

In this study, we used the QTL-seq approach to rapidly identify QTLs controlling rice grain elongation (GE) in an F_2_ population derived from parents differing in GE and other cooking qualities, such as amylose content and gelatinization temperature. The QTLs identified in this study suggest that the candidate genes for GE could be those involved in the starch biosynthetic pathway. The results of this study could be useful for rice breeding for better grain elongation based on the population derived from parental lines with contrasting GT and amylose content. Bulk-segregant analysis together with next-generation sequencing (NGS) could replace the tedious steps of traditional QTL mapping to rapidly identify the major QTL of a complex trait.

## Results

### Mapping population development and plant selection for bulk preparation

**Figure 1:**
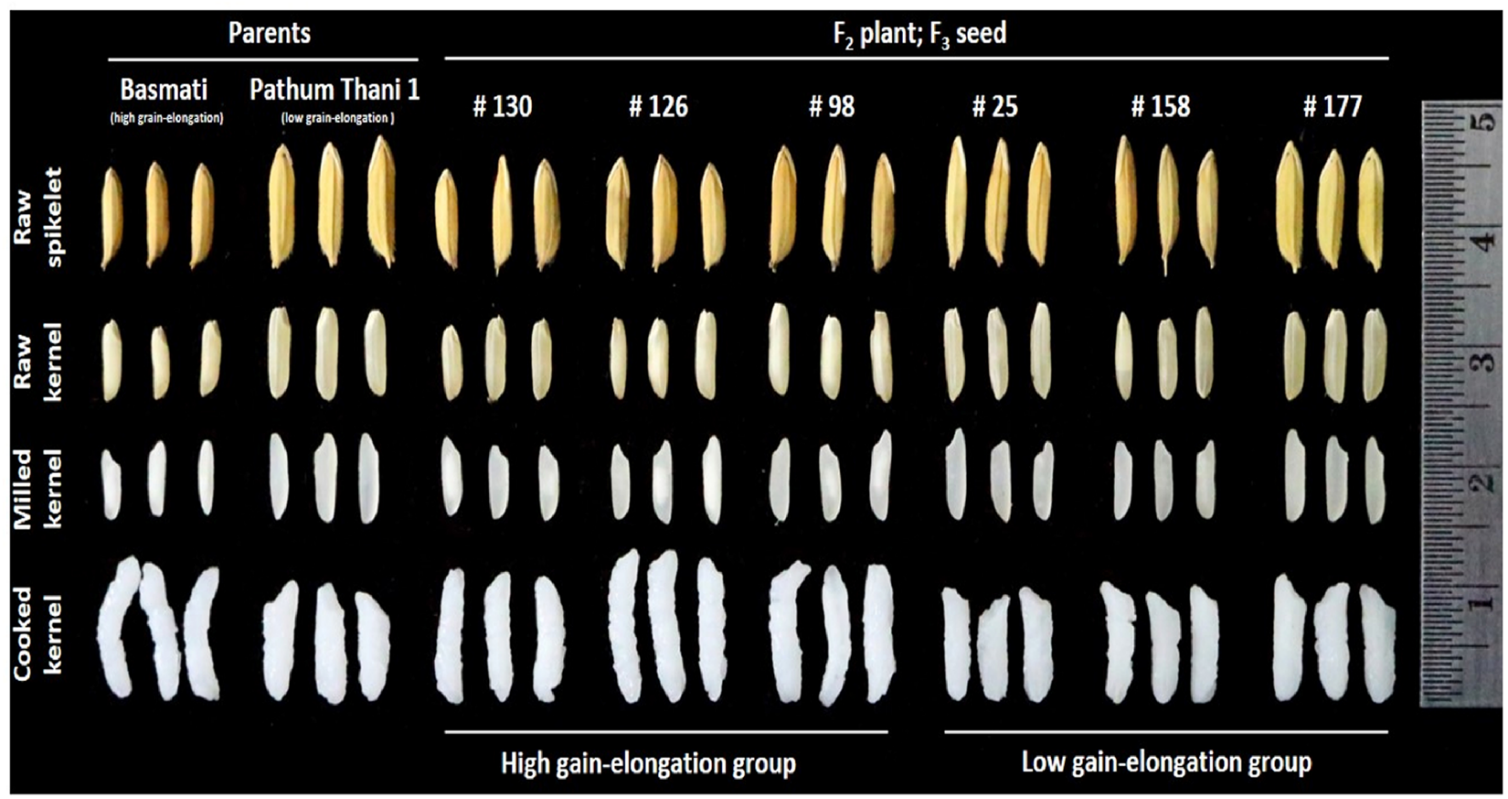
Grain appearance phenotypes and grain elongation of Basmati, PTT1 and some F_2_ lines.

**Figure 2:**
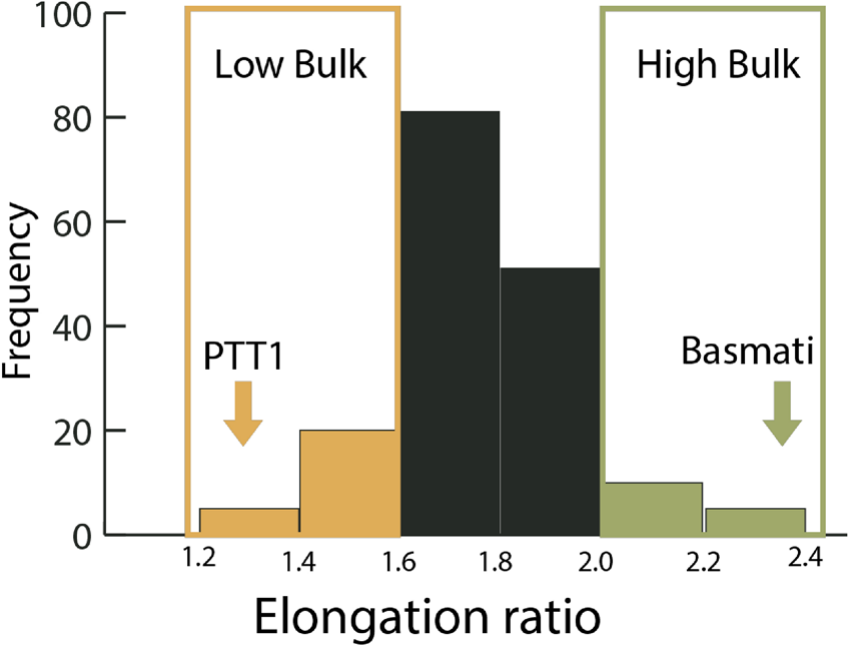
Trait distribution based on grain elongation ratio of the F_2_ population. Arrows indicate the average grain elongation ratio of the two parents. The plants that were selected to build the high and low grain elongation bulks are highlighted in the rectangles.

To rapidly identify QTLs for cooked-grain elongation using the QTL-seq approach, we developed an F_2_ mapping population derived from a cross between Basmati and Pathum Thani 1 (PTT1), a Thai fragrant rice (Fig. 1). Both parental lines are aromatic rice but differ in cooked-grain elongation (GE) ratio and other cooking quality traits, such as the amylose content (AC) and gelatinization temperature (GT). PTT1 contained low AC (16%), low GT (inferred from alkali spreading value (AS) of 5–6) and low GE ratio (1.5), whereas Basmati contained intermediate-high AC (25%), high GT (AS value of 1–2) and high GE ratio (2.2). The average length of milled grains of PPT1 was increased compared with Basmati (7.36 ± 0.15 mm versus 6.38 ± 0.08 mm, respectively). On the contrary, the average length of cooked grains of PTT1 was reduced compared with Basmati (11.09 ± 0.52 mm versus 14.47 ± 0.38 mm, respectively). Hence, the grain elongation (GE) ratio of Basmati was increased compared with PTT1 (2.26 ± 0.06 versus 1.50 ± 0.04, respectively). In the F_2_ progenies, the lengths of milled grains were in the range of 5.90 to 8.88 mm, whereas the lengths of cooked grains were in the range of 9.04 mm to 17.00 mm (Supplementary Fig. S1). The GE ratios of 178 F_2_ lines were between 1.35 and 2.37, and the frequency distribution of the F_2_ lines in different classes of GE ratio was close to normal distribution, suggesting a polygenic mode of inheritance of this trait (Fig. 2 and Supplementary Table S1). In addition to GE, other grain appearance and grain quality traits were segregated among the F_2_ progenies (Fig. 1). However, in this study, we focused only on the GE trait. For QTL-seq analysis, we defined 20 plants with extremely low GE ratios and 20 plants with high GE ratios to generate the low and high GE bulks, respectively (Fig. 2 and Supplementary Table S2).

### Whole-genome resequencing and QTL-seq analysis

**Figure 3:**
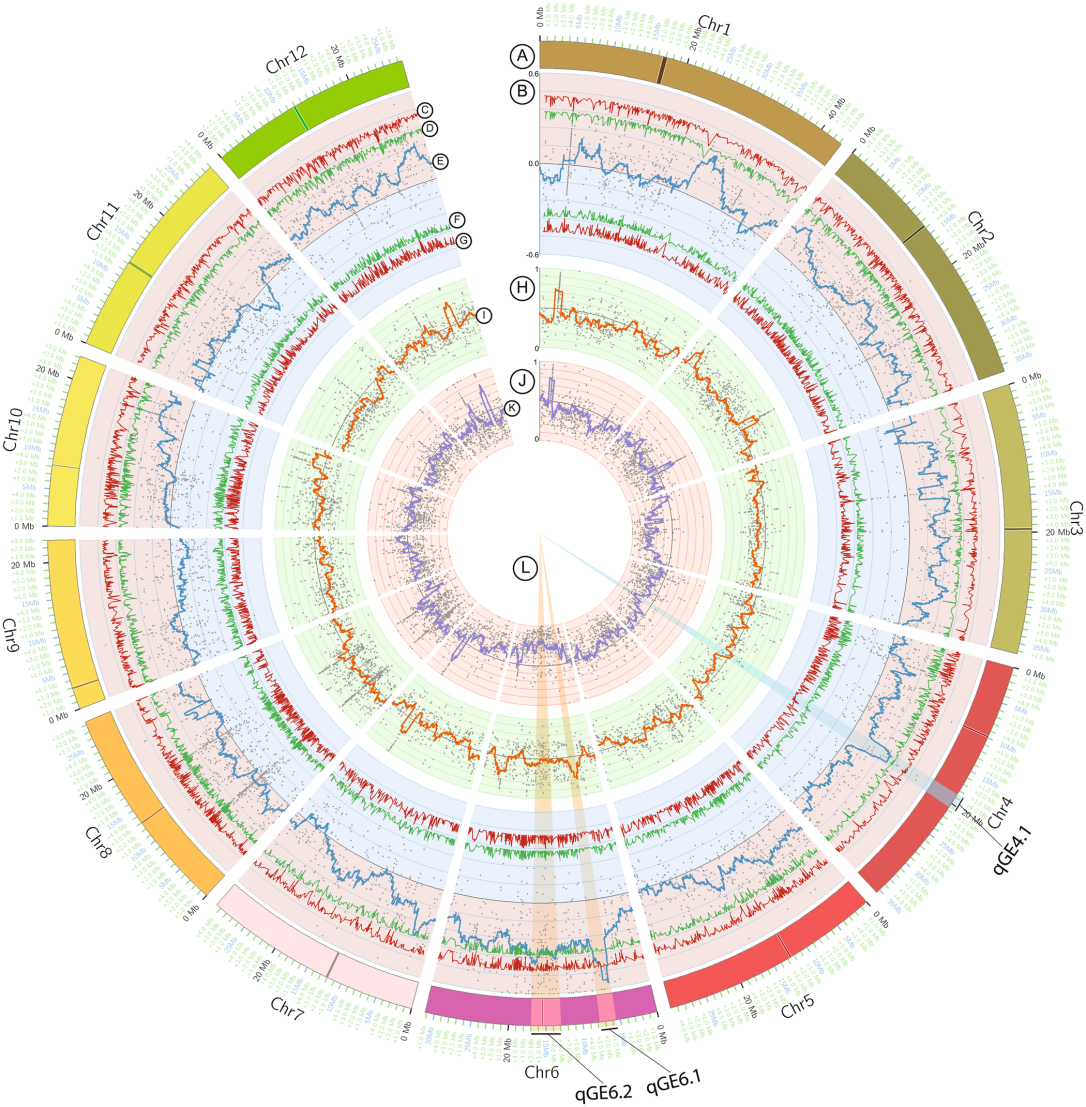
Plots of SNP index of two bulks (HGE bulk and LGE bulk) and ∆(SNP index) compared between them. (**A**) Psuedomolecules of Nipponbare reference genome (IRGSP 1.0). (**B**) Plots of ∆(SNP index) compared between two bulks (HGE bulk and LGE bulk). (**C**) Upper probability values at 99% confidence (P < 0.01). (**D**) Upper probability values at 95% confidence (P < 0.05). (**E**) The sliding window plots of average SNP indexes with a 2-Mb window size and 10-kb steps. (**F**) Lower probability values at 95% confidence (P < 0.05). (**G**) Lower probability values at 99% confidence (P < 0.01). (**H**) SNP index plots of HGE bulk. (**I**) The sliding window plots of average SNP index values with a 2-Mb window size and 10-kb steps. (**J**) SNP index plots of LGE bulk. (**K**) The sliding window plots of average SNP index values with a 2-Mb window size and 10-kb steps. (**L**) Candidate genomic regions containing QTLs for grain elongation.

DNA of high-GE (HGE) and low-GE (LGE) bulks together with the two parents were sequenced the whole genome using Illumina HiSeq. 2500. As a result, 150-bp clean paired-end sequences were generated, yielding 17.0 Gb (113 million reads) for each bulk and 8.5 Gb (56 million reads) for parental lines. The low-quality sequences were filtered to exclusively obtain high-quality sequences of which 90 percent or greater of the individual bases contained Phred scores of 30 or greater. Thus, approximately 60.9, 61.7, 44.6 and 44.2 million reads of paired-end sequences were retained in the HGE-bulk, LGE-bulk, PTT1 and Basmati, respectively, which were equivalent to 21x, 23x, 16x and 16x coverage of the rice genome (~400 Gb) in HGE-bulk, LGE-bulk, PTT1 and Basmati lines (Table 1). The high-quality reads of PTT1 were used to generate the reference sequence of the PTT1 cultivar (see Materials and Methods). By aligning the high-quality reads of the two bulks onto the PTT1 reference sequence, a total of 975,242 SNPs with the support of at least three reads were commonly identified in the two bulks (Table 2). However, to obtain robust results, we exclusively considered SNPs with read supports of at least 29 reads. Thus, 5,466 high confident SNPs were selected to calculate the SNP index in each bulk (Table 2). SNPs with SNP index <0.3 in both bulks were removed as they could be spurious SNPs caused by sequencing errors or alignment errors. The SNP index of remaining SNPs calculated from each bulk was physically plotted throughout 12 rice chromosomes (Fig. 3). The ∆(SNP index) calculated by subtracting the SNP index values in HGE-bulk by those in LGE-bulk together with the sliding windows of average SNP indices of SNPs located within a 2-Mb region and 1-kb stepwise were also plotted (Fig. 3).

**Table 1:** Summary of Illumina sequencing data of parental lines and high and low GE bulks.

Sample	Clean reads	Clean data (Gb)	High^a^-quality reads	High-quality data (Gb)	Average^b^ depth
PTT1	56,882,288	8.53	44,628,822	6.69	16.73
Basmati	56,920,100	8.53	44,294,826	6.64	16.61
High GE bulk	113,902,480	17.0	60,948,512	9.14	21.25
Low GE bulk	113,766,166	17.0	61,772,004	9.26	23.16

^a^The short reads of which 90 percent or above of the individual bases contained the Phred score of 30 or greater.

^b^The average depth of high-quality sequences.

**Table 2:** Chromosome-wise distribution of single nucleotide polymorphisms (SNPs) between the two pools.

Chromosome	Length	Total number of SNPs (depth > = 3)	Selected SNPs (depth > = 29)
1	43,270,923	110,255	564
2	35,937,250	112,971	572
3	36,413,819	71,795	306
4	35,502,694	76,962	430
5	29,958,434	77,046	442
6	31,248,787	85,383	452
7	29,697,621	59,361	301
8	28,443,022	92,078	737
9	23,012,720	70,213	362
10	23,207,287	69,774	396
11	29,021,106	74,449	442
12	27,531,856	74,955	462
Total	373,245,519	975,242	5,466

### Candidate genomic regions for cooked grain elongation

**Figure 4:**
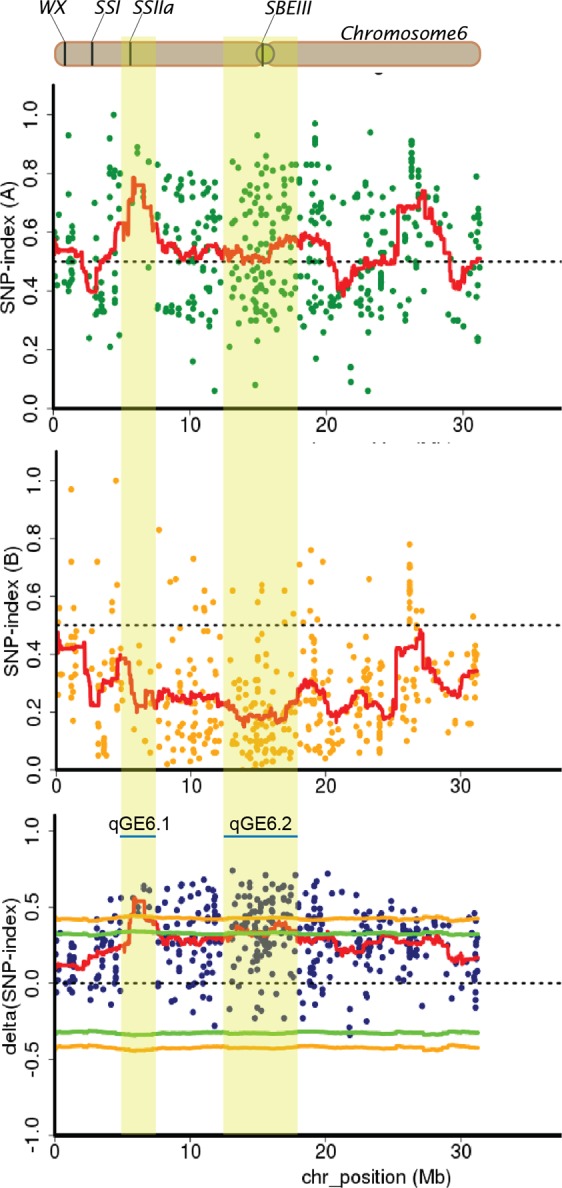
SNP index plots between two bulks and ∆(SNP index) demonstrating the genomic region with differing SNP indexes in two bulks. The identified QTL regions on chromosome 6 (qGE6.1 and qGE6.2) are highlighted.

Based on the SNP index plots of the HGE and LGE bulks and the plots of ∆(SNP index), we identified three candidate genomic regions for GE, two regions on chromosome 6 and one region on chromosome 4 (Fig. 3 and Table 3). One of the genomic regions identified on chromosome 6 between 5.59 and 7.70 Mb, namely *qGE6.1*, exhibited the highest contrasting patterns of SNP index graphs for HGE and LGE bulks (Fig. 4). The plants in HGE bulk mainly had Basmati genomic segments in this region (the average SNP index of 0.78), whereas the plants in LGE bulk mainly had the PTT1-type genome (the average SNP index of 0.22). The ∆(SNP index) plots at this region were the highest and mainly above the statistical confidence intervals for this read depth (statistical significance under the null hypothesis: P < 0.01). The location of *qGE6.1* was on the short arm of chromosome 6 in the proximity to a few known endosperm starch biosynthetic genes, i.e., *granule-bound starch synthase I (GBSSI)* or *Wx (*LOC_Os06g04200), *starch synthase I* (*SSI:* LOC_Os06g06560) *and starch synthase IIa* (*SSIIa:* LOC_Os06g12450) (Fig. 4). However, *Wx* and *SSI* were located toward the far end of the short arm of chromosome 6, which is outside the QTL region. On the other hand, *SSIIa* (6:6,748,358-6,753,338) was located within *qGE6.1* and close to the peak region (Fig. 4). Approximately 278 genes were located within 1 megabase (Mb) region encompassing *qGE6.1* (Supplementary Table S3). Thirteen of the 64 genes within 500 kb surrounding the peak of *qGE6.1* contained nonsynonymous SNPs in the coding sequences (CDSs) compared between the two parents (Table 4). However, among these genes, only *SSIIa* is known to be involved in rice endosperm starch biosynthesis.

**Table 3:** Summary of QTLs detected for cooked grain elongation ratio.

QTL	Chr.	Location		Interval (Mb)	Delta (SNP index)		Confidence interval		Donor
		Start	End		Min	Max	95%	99%	
qGE4.1	4	18,530,000	20,570,000	2.04	0.30	0.31	0.33	0.43	Basmati
qGE6.1	6	5,590,000	7,770,000	2.18	0.31	0.56	0.33	0.43	Basmati
qGE6.2	6	13,000,000	17,000,000	4.00	0.31	0.41	0.33	0.43	Basmati

**Table 4:** List of predicted candidate genes in 500-Kb intervals of the qGE6.1 region.

Chr	Start	Stop	Gene ID	No. of missense SNPs	Putative function
6	6515548	6519199	LOC_Os06g12170	1	Expressed protein
6	6521987	6523338	LOC_Os06g12180	1	Jacalin-like lectin domain containing protein
6	6682411	6685947	LOC_Os06g12330	1	Amino acid transporter
6	6696666	6700782	LOC_Os06g12360	1	Pentatricopeptide
6	6736053	6737803	LOC_Os06g12410	1	GDSL-like lipase/acylhydrolase,
6	6758181	6762995	LOC_Os06g12460	1	CSLA3 - cellulose synthase-like family A; mannan synthase
6	6503735	6514399	LOC_Os06g12160	2	AAA-type ATPase family protein
6	6570778	6572727	LOC_Os06g12230	2	TCP-domain protein, putative
6	6754294	6755541	LOC_Os06g12455	2	Expressed protein
6	6595940	6602476	LOC_Os06g12260	3	N-rich protein, putative
6	6715886	6722017	LOC_Os06g12390	3	Galactosyltransferase family protein
6	6484043	6488553	LOC_Os06g12120	4	BRASSINOSTEROID INSENSITIVE 1-associated receptor kinase 1 precursor
6	6748358	6753338	LOC_Os06g12450	4	Soluble starch synthase 2–3, chloroplast precursor

Another genomic region identified on chromosome 6 with ∆(SNP index) plots greater than the statistical confidence intervals (P < 0.05), namely *qGE6.2*, was located in the 13.0- to 17.0-Mb region flanking the centromere (at 15.3 Mb). This region is a compound region composed of two smaller regions peaking at 13.5 and 16.5 Mb, separately (Fig. 4). Although ∆(SNP index) in this region is also high, the average SNP index in HGE bulk (the average SNP index of 0.55) is reduced compared with the *qGE6.1* region, suggesting that an equal amount of Basmati and PTT1 genomic segments are present in the HGE bulk. However, the LGE bulk plants mainly had a PTT1-type genome (the average SNP index of 0.18). Nevertheless, no known gene related to starch biosynthesis was identified within the 1-Mb region flanking the two small peaks of *qGE6.2* (Supplementary Table S4), but a *1,4-alpha-glucan*-*branching enzyme* or *SBEIII* (LOC_Os06g26234) was identified in the middle region of *qGE6.2*, which was close to the centromeric region of rice chromosome 6 (at 15.3 Mb).

**Figure 5:**
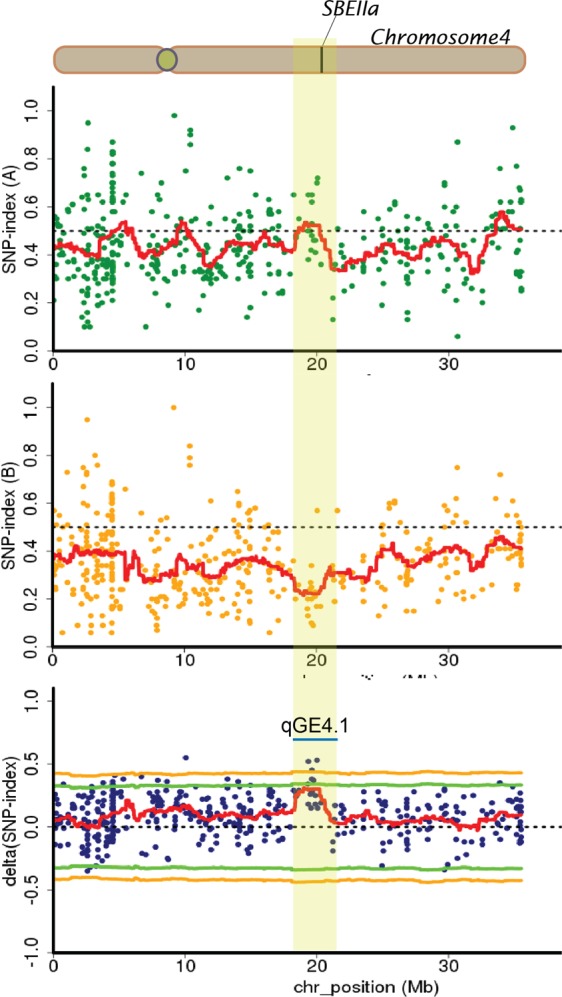
SNP index plots between two bulks and ∆(SNP index) demonstrating the genomic region with differing SNP indexes in two bulks. The identified QTL region on chromosome 4 (qGE4.1) is highlighted.

The genomic region identified on chromosome 4, namely *qGE4.1*, was located between 18.53 and 20.57 Mb (Table 3). The average SNP index of the SNPs was approximately 0.5 in the HGE bulk and approximately 0.2 in the LGE bulk at this region. Although the ∆(SNP index) of this QTL was not statistically significant given that the peak of sliding window plots was less than the statistical confidence interval, the annotated genes within the QTL included the starch branching enzyme *SBEIIa* (LOC_Os04g33460), which is the gene involved in amylopectin biosynthesis (Fig. 5, Supplementary Table S5).

### Validation and confirmation of the identified genomic regions on chromosomes 4 and 6

We chose to examine *SSIIa* as a candidate gene representing *qGE6.1*, *SBEIII* as a candidate gene representing *qGE6.2* and *SBEIIa* as a candidate gene representing *qGE4*. A KASP maker developed for *SSIIa* based on the two consecutive SNPs (GC/TT) at the position 6,752,887-6,752,888, a CAPS marker developed based on a SNP (C/T) at the position 15,350,350 on exon 11 of *SBEIII* (LOC_Os06g26234) and a KASP marker developed based on a SNP (C/T) of *SBEIIa* at the position 20,241,818 were used to validate the 40 individual plants in HGE and LGE pools. The ratio of the frequency of Basmati’s allele to the frequency of PTT1’s allele in the HGE pool revealed by the *SSIIa* marker was 0.75:0.25, that revealed by the *SBEIII* marker was 0.57:0.43, and that revealed by the *SBEIIa* marker was 0.53:0.47 (Supplementary Table S6). The ratio of the frequency of Basmati’s allele to the frequency of PTT1’s allele in the LGE pool revealed by *SSIIa* was 0.20:0.80, that revealed by *SBEIII* was 0.15:0.85, and that revealed by *SBEIIa* was 0.27:0.73 (Supplementary Table S6). These allele frequencies followed a similar pattern of the SNP index in the two pools identified by QTL-seq analysis.

The *SSIIa*, *SSI*, *Wx*, *SBEIII* and *SBEIIa* markers were genotyped for the 170 F_2_ plants, and single marker analysis was performed to validate the association of the markers with cooked-grain elongation phenotypes (Supplementary Table S7). As a result, *SSIIa* explained 18.58% of phenotypic variation (PVE) with a LOD score of 7.99 (Table 5), whereas the *SBEIII* marker explained 11.34% of phenotypic variation with a LOD score of 4.93. Surprisingly, the *SSI* marker was also significant with a LOD score of 4.35, and it had the PVE of 9.91%. The *Wx* marker and the *SBEIIa* marker were not significant for the grain elongation ratio given that the LOD score was less than 3. Collectively, these QTLs explained 20.14% of the total phenotypic variation. The positive alleles of grain elongation of all markers were derived from Basmati.

**Table 5:** Single marker analysis of the three markers for *Wx*, *SSI*, *SSIIa, SBEIII* and *SBEIIa* and grain elongation ratio of the F2 population.

Marker	Chromosome	LOD	PVE(%)	Additive effect	Donor
Wx	6	2.33	4.88	0.050	Basmati
SSI	6	4.35	9.91	0.075	Basmati
SSIIa	6	7.99	18.58	0.105	Basmati
SBEIII	6	4.93	11.34	0.084	Basmati
SBEIIa	4	2.22	4.48	0.054	Basmati
Total			20.14		

## Discussion

Cooked grain elongation is an important characteristic of rice-cooking quality that is influenced by starch properties. The trait could be measured by the ratio of grain elongation (cooked grain length ratio to milled grain length) and by the grain elongation index (a proportionate rice grain change after cooking)^6^. In this study, we measured the grain elongation ratio to determine cooked grain elongation. The main focus of this study was to utilize the QTL-seq approach to locate QTLs for grain elongation in an F_2_ population derived from the two parents (Basmati and PTT1) differing in grain elongation and other cooking quality traits, i.e., amylose content and gelatinization temperature. QTL-seq has been used previously to identify QTL in many crops including rice as it could replace the tedious steps of traditional QTL mapping to rapidly identify the major QTL^15^. In this study, we identified three QTLs for the cooked-grain elongation trait in rice. Two QTLs were located on chromosome 6 (*qGE6.1* and *qGE6.2*), and another QTL was located on chromosome 4 (*qGE4.1*). Among these, *qGE6.1* was the most statistically significant. All three detected QTLs were located near the locations of known starch biosynthesis genes. For example, the QTLs on chromosome 6, *qGE6.1* and *qGE6.2*, were located near *SSIIa* and *SBEIII*, respectively, and the QTL on chromosome 4, *qGE4.1*, was located near *SBEIIa*. According to the marker-trait analysis results, the SNPs in *SSIIa* and *SBEIII* exhibited an association with the grain elongation phenotype. However, the *SBEIIa* marker on chromosome 4 showed no strong association with the phenotype compared to *SSIIa* and *SBEIII* markers. Although these three genes could be potential candidates given that they are involved in starch biosynthesis, fine mapping is still required to identify the causal genes within each QTL.

The association between gelatinization temperature (GT) and grain elongation (GE) has been mentioned in a previous study wherein GE was considered a physical phenomenon and influenced by GT^5^. GT in rice is mainly controlled by *starch synthase IIa (SSIIa)*, which is located on chromosome 6^21^. In the present study, we identified the gene for gelatinization temperature *SSIIa* as a candidate gene in the *qGE6.1*. This finding is also supported by a previous study demonstrating that one main effect QTL is associated with the grain elongation near *SSIIa* (*Alk*) locus based on 86 doubled haploid lines derived from IR64 and Azucena, which are different in GT but exhibit similar amylose content^22^. GE might be similar to other complex traits that are controlled by multiple loci, many of which are small effect QTLs^23^. According to the single-marker analysis result, the effect of *qGE6.1* was relatively minor given that only 18% of the phenotypic variance was explained using the marker specific to *SSIIa*. A previous study also identified a QTL within the region overlapping with *qGE6.1* as located between RM276-RM549 (6.23–6.97 Mb) with a similar percentage of the explained phenotypic variance^10^.

High grain elongation in Basmati may also be influenced by the structural arrangement of starch molecules in the endosperm. Starch in rice endosperm is composed of amylose and amylopectin. Several genes encoding various isoforms of several enzymes, including starch synthases and starch branching enzymes, are involved in starch biosynthesis in rice grains. Starch synthases include granule-bound starch synthase (GBSS) or Wx, which is involved in amylose biosynthesis, and soluble starch synthase classes, i.e., SSI, SSII, SSIII and SSIV, which are involved in amylopectin synthesis. Several *SBE* genes are present in plants, and various SBE isoforms impact the structural and functional properties of starch^24^. A *starch synthase (SSIIa)* and two *starch-branching enzymes (SBEIIa and SBEIII)*, which encode enzymes involved in amylopectin biosynthesis, were identified in each QTL in this study, whereas *Wx* or *granule-bound starch synthase I (GBSSI)*, which is responsible for amylose biosynthesis, was not included in the detected QTLs. Although amylose content is positively correlated with elongation ratio^25^ and QTLs encompassing the *Wx* locus influencing cooked grain elongation were also reported^2,26^, the *Wx-*KASP marker, which could determine *Wx*^*a*^ and *Wx*^*b*^ alleles, was not significantly associated with grain elongation ratio based on this population given that the parental lines Basmati and PTT1 exhibited contrasting amylose content as they contained *Wx*^*a*^ and *Wx*^*b*^, respectively. This finding potentially suggests the role of amylopectin rather than amylose in association with grain elongation based on this type of population.

Among three types of SBE isoforms (SBEI, SBEII and SBEIII) that exist in higher plants, SBEIII has been minimally studied due to the difficulty in isolating and purifying the encoded protein of the gene^27,28^. Given that *SBEIII* identified within *qGE6.2* was located in the centromeric region, where expression of genes is largely suppressed^28,29^, expression analysis of this gene and its involvement in endosperm starch biosynthesis should be confirmed. *SBEIIa* is one of the two isoforms present in rice and other cereals. The two isoforms SBEIIa and SBEIIb exhibit distinct expression patterns. The expression of SBEIIb is restricted to the endosperm, whereas the expression of SBEIIa is versatile and comparatively reduced^30^. The interaction between these genes as well as other starch biosynthesis genes and their effects on grain elongation requires further investigation. Given that the two parental lines exhibit different starch properties, progenies with different starch profiles and high grain-elongation could be selected from this population and will be useful for developing high-quality rice. Given its high grain elongation characteristic, Basmati rice is a good source for elucidating the genetic control of GE and improving rice with higher grain elongation in different genetic backgrounds. The markers developed based on functional variations within these genes could be useful for rice breeding programs for high grain elongation using the population derived from Basmati x non-basmati crosses.

## Materials and Methods

### Development of F_2_ population segregating for grain elongation

An accession of Basmati rice was selected as the high grain-elongation (GE) parent and an elite Thailand’s aromatic rice variety, Pathum Thani 1 (PTT1), was selected as the low GE parent to generate a segregating population for cooked-grain elongation. Crosses between PTT1 and Basmati were made using an emasculation method to generate F_1_ seeds. The F_1_ seeds were grown in pots in the greenhouse and self-pollinated at Rice Science Center, Kasetsart University, Nakhon Pathom, Thailand. The F_2_ seeds were collected from a few self-pollinated F_1_ plants and grown to generate an F_2_ population with few hundreds of progenies. Approximately 200 F_2_ plants were grown and self-pollinated to produce F_3_ seeds, which were used to evaluate the phenotype.

### Evaluation of grain elongation

Thirty random milled grains of each F_2:3_ family derived from 178 F_2_ lines were collected and evaluated the linear grain elongation ratio (ratio of mean length of cooked and raw grains) using the method previously described^6^. The evaluation was performed in three replications using ten grains per replication. Trait inheritance was determined based on the distribution of the F_2_ phenotypes.

### Sample pooling, DNA isolation and whole-genome resequencing

QTL-seq analysis was performed by collecting two groups of the F_2_ lines with distinctive phenotypes, such as high grain-elongation (HGE) and low grain-elongation (LGE) ratios. Forty F_2_ plants were selected to generate HGE and LGE bulks, each with twenty plants. Leaf samples were collected from each F_2_ plant, and genomic DNA was individually isolated using the DNeasy Plant Mini Kit (QIAGEN, USA). DNA samples from 20 plants with extremely high GE ratios were mixed with equal amounts and used as HGE-pool, and DNA samples of 20 plants with extremely low GE ratios were mixed with equal amounts and used as LGE-pool. DNA-seq libraries were constructed from the genomic DNA of the two pools as well as from that of the two parents. DNA-seq libraries were sequenced the whole genome using Illumina HiSeq. 2500 platform (Illumina, Inc., USA) to generate paired-end read data with a sequencing depth of approximately 40x of the rice genome (~400 Mb) for each pool and 20x for parental plants.

### Read mapping, SNP calling and SNP index analysis

The raw sequencing data were filtered according to strict parameters to obtain high-quality data. QTL-Seq analysis was performed using the QTL-seq pipeline as described previously^15^. At the beginning of the analysis, the sequencing data of either parent was required to generate a reference genome of the parent to be used as the reference for read mapping of the two bulk samples. In this study, we used PTT1 as a reference throughout the entire analysis pipeline. First, the clean reads of the PTT1 parent were aligned to the public reference genome (Nipponbare: IRGSP1.0) using BWA aligner^31^. The variants representing the PTT1 parent were then used to develop the PTT1 reference genome by substituting the bases in the genome. DNA variants, including single nucleotide polymorphism (SNP) and small insertion/deletion (Indel), were detected in the HGE and LGE bulks by aligning reads onto the PTT1 reference genome. Then, the SNP index at each SNP position was calculated for the HGE and LGE bulks as described previously^32^. The SNP positions with SNP index <0.3 in both pools were removed. The ∆(SNP index) was then calculated using the following formula: [SNP index (HGE bulk) – SNP index (LGE bulk)]. The distribution of average SNP index and ∆(SNP index) was estimated in a given genomic interval using a sliding window approach with a 2-Mb window size and 10-kb step and plotted to generate SNP index plots for all rice chromosomes. To make the plots less complex, only the SNPs with read supports of 29 or greater were selected. The plots of SNP index and ∆(SNP index) compared between the two bulks were visualized using Circos^33^. The candidate genomic regions for grain elongation were determined based on the sliding window plots. Only the regions in which the average ∆(SNP index) of a locus was significantly greater than the surrounding region and windows that exhibited an average P-value < 0.05 were considered^15^.

### Identification of nonsynonymous SNPs in QTL regions

Whole-genome resequencing data of the two parents, PTT1 and Basmati, were used to identify nonsynonymous SNPs. The data were aligned against the Nipponbare reference genome using BWA aligner. SNPs were identified with BAM files obtained in the previous step using Samtools 1.0^34^. The effects of the obtained SNPs were annotated using Variant Effect Predictor (VEP: https://plants.ensembl.org/Oryza_sativa/Tools/VEP). Nonsynonymous SNPs and other SNPs with strong effects present in the candidate genomic regions identified by QTL-seq were selected.

### Marker genotyping and marker-trait association analysis

The pre-designed Kompetitive Allele Specific PCR (KASP) markers for *SSIIa, SBEIIa, Wx* and *SSI* and a CAPS marker for *SBEIII* were used to genotype the F_2_ progenies. *SSIIa*-KASP (unpublished) was designed based on the two consecutive functional SNPs (TT/GC) on exon 8 to detect specific alleles. *Wx*-KASP (unpublished) was designed based on the SNP (G/T) associated with *Wx*^*a*^/*Wx*^*b*^ alleles located at the 5′ splicing site of intron 1 of the *Wx*. *SSI*-KASP (unpublished) was designed based on a nonsynonymous SNP (G/A) at position 3,070,895 on exon 14 of *SSI*. *SBEIIa*-KASP (unpublished) was designed based on a SNP (C/T) at position 20,241,818 on intron 1. The *SBEIII*-CAPS marker (unpublished) was designed to detect the SNP (C/T) at the position 15,350,350 on exon 11 of *SBE-III* (LOC_Os06g26234). All markers were provided courtesy of Rice Gene Discovery, BIOTEC, Thailand. Single-marker analysis was performed using the genotype data from the four markers and the phenotypic data of 170 F_2_ lines. The percentage of phenotypic variance explained by each QTL (R^2^) was estimated by simple regression analysis.

## Supplementary information

Supplementary Information
