# Supplementary material for: QTL-seq identifies cooked grain elongation QTLs near soluble starch synthase and starch branching enzymes in rice (Oryza sativa L.)
Source: Sci Rep. 2019 Jun 6;9:8328. doi: 10.1038/s41598-019-44856-2 (PMC6554297; doi:10.1038/s41598-019-44856-2)
Supplement: Supplementary file 1 — Supplementary Information [file 41598_2019_44856_MOESM1_ESM.pdf]

QTL-seq identifies cooked grain elongation QTLs near *soluble starch synthase* and *starch branching enzymes* in rice (*Oryza sativa* L.)

Siwaret Arikit[1,2], Samart Wanchana[3], Srisawat Khanthong[4], Chatree Saensuk[2], Tripop Thianthavon[4], Apichart Vanavichit[1,2] & Theerayut Toojinda[3*]

1. Department of Agronomy, Faculty of Agriculture at Kamphaeng Saen, Kasetsart University Kamphaeng Saen Campus, Nakhon Pathom, 73140, Thailand
2. Rice Science Center, Kasetsart University Kamphaeng Saen Campus, Nakhon Pathom, 73140, Thailand
3. National Center for Genetic Engineering and Biotechnology (BIOTEC), National Science and Technology Development Agency (NSTDA), Khlong Luang, Pathum Thani 12120, Thailand
4. Faculty of Agriculture at Kamphaeng Saen, Kasetsart University Kamphaeng Saen Campus, Nakhon Pathom 73140, Thailand

* Corresponding author at: National Center for Genetic Engineering and Biotechnology (BIOTEC), National Science and Technology Development Agency (NSTDA), Khlong Luang, Pathum Thani 12120, Thailand

E-mail addresses: theerayut@biotec.or.th (Theerayut Toojinda)

Supplementary Fig. S1

Box plots of average raw grain length (A.) and cooked grain length (B.) among 178 F2 lines. The positions of the grain lengths in Pathum Thani1 (PTT1) and Basmati are presented with their names.

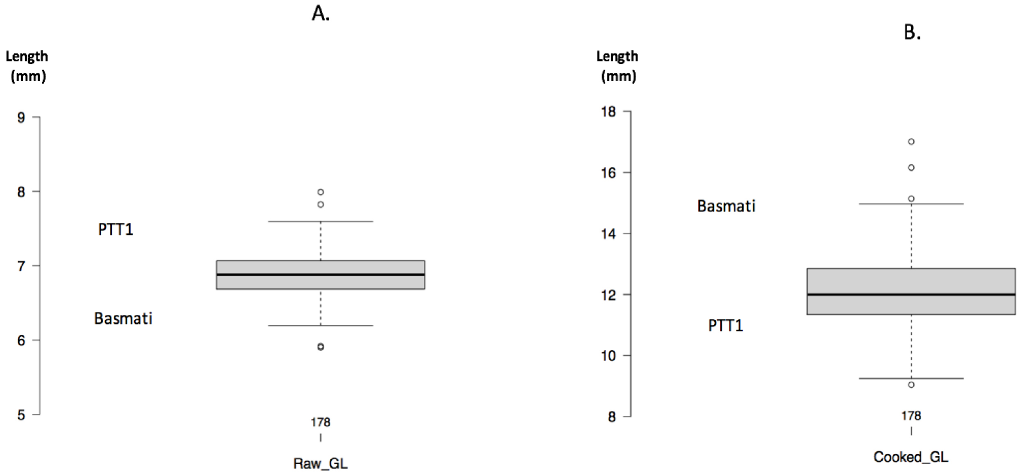

Supplementary Table S1 Phenotypic data analyses of grain elongation ratio for 178 F_2 individuals.

Trait	PTT1	Basmati	F2 Population				
			Mean	Max	Min	Skewness	Kurtosis
GE	1.50	2.26	1.76	2.37	1.35	0.46	3.8

Supplementary Table S2 Forty selected F_2 lines used to generate high GE bulk and low GE bulk.

Low GE bulk		High GE bulk	
Line number	Average GE	Line number	Average GE
56	1.46	37	1.88
168	1.49	71	1.88
100	1.51	98	1.9
25	1.53	79	1.91
108	1.53	35	1.93
19	1.54	8	1.93
184	1.55	52	1.94
156	1.56	179	1.95
40	1.57	95	1.95
6	1.57	137	1.96
120	1.58	101	1.98
169	1.58	126	1.99
176	1.58	148	2.03
177	1.6	89	2.03
83	1.6	149	2.04
196	1.61	73	2.05
22	1.61	139	2.08
153	1.62	5	2.1
161	1.63	130	2.13
18	1.64	118	2.18

Supplementary Table S3 List of predicted candidate genes in 1-Mb intervals of the qGE6.1 region.

Chr	Start	Stop	Gene Name	Putative Function
6	5497369	5498771	LOC_Os06g10590	expressed protein
6	5499342	5502411	LOC_Os06g10600	homeobox and START domains containing protein, putative, expressed
6	5522553	5523176	LOC_Os06g10610	expressed protein
6	5523780	5528576	LOC_Os06g10620	transcription elongation factor SPT5 homolog 1, putative, expressed
6	5555007	5559140	LOC_Os06g10650	tyrosine phosphatase family protein, putative, expressed
6	5559909	5563097	LOC_Os06g10660	lysM domain-containing GPI-anchored protein 1 precursor, putative,
6	5569910	5571471	LOC_Os06g10670	aspartic proteinase nepenthesin-1 precursor, putative, expressed
6	5572599	5573850	LOC_Os06g10680	expressed protein
6	5575867	5584373	LOC_Os06g10690	PHD-finger domain containing protein, putative, expressed
6	5586611	5586901	LOC_Os06g10700	expressed protein
6	5588196	5594757	LOC_Os06g10710	expressed protein
6	5597520	5597825	LOC_Os06g10720	expressed protein
6	5601748	5604984	LOC_Os06g10730	expressed protein
6	5613999	5617153	LOC_Os06g10750	integral membrane protein DUF6 containing protein, expressed
6	5619105	5621750	LOC_Os06g10760	emp24/gp25L/p24 family protein, putative, expressed
6	5621926	5625701	LOC_Os06g10770	peptidase, putative, expressed
6	5629416	5631997	LOC_Os06g10780	AP2 domain containing protein, expressed
6	5633354	5635721	LOC_Os06g10790	lectin-like receptor kinase, putative, expressed
6	5636936	5637931	LOC_Os06g10800	zinc finger family protein, putative, expressed
6	5638272	5641660	LOC_Os06g10810	phosphate carrier protein, mitochondrial precursor, putative, expressed
6	5642245	5642880	LOC_Os06g10820	helix-loop-helix DNA-binding domain containing protein, expressed
6	5653538	5653942	LOC_Os06g10840	expressed protein
6	5657850	5661265	LOC_Os06g10850	lipase, putative, expressed
6	5663968	5665309	LOC_Os06g10860	glucosyltransferase, putative, expressed
6	5677080	5682126	LOC_Os06g10880	bZIP transcription factor, putative, expressed
6	5688535	5689411	LOC_Os06g10890	sterol carrier protein-2, putative, expressed
6	5693807	5694649	LOC_Os06g10900	DELLA protein RGL3, putative, expressed

6	5701639	5704991	LOC_Os06g10910	xyloglucan fucosyltransferase, putative, expressed
6	5705127	5707910	LOC_Os06g10920	xyloglucan fucosyltransferase, putative, expressed
6	5712154	5714660	LOC_Os06g10930	xyloglucan fucosyltransferase, putative, expressed
6	5717068	5717478	LOC_Os06g10940	expressed protein
6	5718733	5720698	LOC_Os06g10950	xyloglucan fucosyltransferase, putative, expressed
6	5721756	5726745	LOC_Os06g10960	xyloglucan fucosyltransferase, putative, expressed
6	5732121	5734237	LOC_Os06g10970	xyloglucan fucosyltransferase, putative, expressed
6	5734288	5736153	LOC_Os06g10980	xyloglucan fucosyltransferase, putative, expressed
6	5745188	5747209	LOC_Os06g10990	DnaK family protein, putative, expressed
6	5759685	5761518	LOC_Os06g11010	eukaryotic aspartyl protease domain containing protein, expressed
6	5775433	5777316	LOC_Os06g11020	tic22-like family domain containing protein, expressed
6	5778761	5779333	LOC_Os06g11030	EF hand family protein, putative, expressed
6	5784166	5788998	LOC_Os06g11040	expressed protein
6	5791466	5795810	LOC_Os06g11050	cyclin-dependent kinase inhibitor, putative, expressed
6	5800188	5800857	LOC_Os06g11060	expressed protein
6	5807269	5811211	LOC_Os06g11070	expressed protein
6	5812591	5813000	LOC_Os06g11080	hypothetical protein
6	5813468	5815196	LOC_Os06g11090	CXE carboxylesterase, putative, expressed
6	5830906	5833223	LOC_Os06g11120	expressed protein
6	5835562	5837122	LOC_Os06g11130	gibberellin receptor GID1L2, putative, expressed
6	5837564	5838523	LOC_Os06g11135	gibberellin receptor GID1L2, putative, expressed
6	5840075	5844550	LOC_Os06g11140	pyridine nucleotide-disulphide oxidoreductase domain containing
6	5847981	5849957	LOC_Os06g11150	DUF1645 domain containing protein, putative, expressed
6	5854313	5854669	LOC_Os06g11160	expressed protein
6	5857235	5861126	LOC_Os06g11170	formin-binding protein-related, putative, expressed
6	5862163	5869596	LOC_Os06g11180	OsPOP12 - Putative Prolyl Oligopeptidase homologue, expressed
6	5869895	5876421	LOC_Os06g11190	OsPOP13 - Putative Prolyl Oligopeptidase homologue, expressed
6	5878572	5880549	LOC_Os06g11200	12-oxophytodienoate reductase, putative, expressed
6	5884474	5886141	LOC_Os06g11210	12-oxophytodienoate reductase, putative, expressed
6	5892688	5894037	LOC_Os06g11230	expressed protein

6	5894721	5896352	LOC_Os06g11240	12-oxophytodienoate reductase, putative, expressed
6	5905264	5906018	LOC_Os06g11260	12-oxophytodienoate reductase 2, putative, expressed
6	5908095	5909468	LOC_Os06g11270	anthocyanidin 3-O-glucosyltransferase, putative, expressed
6	5912374	5914368	LOC_Os06g11280	12-oxophytodienoate reductase, putative, expressed
6	5918106	5919690	LOC_Os06g11290	12-oxophytodienoate reductase, putative, expressed
6	5921695	5923664	LOC_Os06g11300	CRR4, putative, expressed
6	5921977	5925597	LOC_Os06g11304	expressed protein
6	5927194	5928879	LOC_Os06g11308	hypothetical protein
6	5930399	5931984	LOC_Os06g11310	plastocyanin-like domain containing protein, putative, expressed
6	5933788	5939645	LOC_Os06g11320	peptidyl-prolyl cis-trans isomerase CYP40, putative, expressed
6	5953593	5963184	LOC_Os06g11330	OsMADS55 - MADS-box family gene with MIKCc type-box, expressed
6	5975645	5976459	LOC_Os06g11360	expressed protein
6	5977297	5980829	LOC_Os06g11370	mediator of RNA polymerase II transcription subunit 6, putative,
6	5982669	5987671	LOC_Os06g11380	geminivirus Rep-interacting motor protein, putative, expressed
6	6003866	6004179	LOC_Os06g11390	expressed protein
6	6007444	6010097	LOC_Os06g11400	expressed protein
6	6019597	6021861	LOC_Os06g11410	cyclin, putative, expressed
6	6027823	6029445	LOC_Os06g11420	SKIP/SNW domain containing protein, expressed
6	6032410	6035429	LOC_Os06g11430	GRAM domain containing protein, expressed
6	6058726	6060541	LOC_Os06g11450	zinc finger, C3HC4 type domain containing protein, expressed
6	6081889	6083447	LOC_Os06g11480	hypothetical protein
6	6086289	6097662	LOC_Os06g11500	MCM9 - Putative minichromosome maintenance MCM family subunit 9,
6	6086798	6088248	LOC_Os06g11490	plastocyanin-like domain containing protein, putative, expressed
6	6101061	6102117	LOC_Os06g11510	expressed protein
6	6102123	6108979	LOC_Os06g11520	LMBR1 integral membrane protein, putative, expressed
6	6141610	6142769	LOC_Os06g11580	expressed protein
6	6144586	6145404	LOC_Os06g11590	expressed protein
6	6146721	6150348	LOC_Os06g11600	growth regulator related protein, putative, expressed
6	6152018	6153438	LOC_Os06g11610	heat shock 22 kDa protein, mitochondrial precursor, putative, expressed
6	6155940	6159888	LOC_Os06g11620	RNA recognition motif containing protein, putative, expressed

6	6160326	6163685	LOC_Os06g11630	OsFBX192 - F-box domain containing protein, expressed
6	6171585	6176329	LOC_Os06g11640	serine/threonine-protein phosphatase 2A activator 2, putative, expressed
6	6177853	6179163	LOC_Os06g11650	phosphate-induced protein 1 conserved region domain containing protein, expressed
6	6180308	6181639	LOC_Os06g11660	phosphate-induced protein 1 conserved region domain containing protein, expressed
6	6189101	6189358	LOC_Os06g11670	expressed protein
6	6191531	6192496	LOC_Os06g11680	phosphate-induced protein 1 conserved region domain containing protein, expressed
6	6199474	6200741	LOC_Os06g11700	phosphate-induced protein 1 conserved region domain containing protein, expressed
6	6202140	6202790	LOC_Os06g11710	cytokinin-O-glucosyltransferase 2, putative, expressed
6	6205607	6207653	LOC_Os06g11720	cytokinin-O-glucosyltransferase 2, putative, expressed
6	6208293	6214438	LOC_Os06g11730	RNA recognition motif containing protein, putative, expressed
6	6222556	6224681	LOC_Os06g11740	OsAPRL2 adenosine 5'-phosphosulfate reductase-like OsAPRL2, expressed
6	6227813	6229557	LOC_Os06g11750	hypothetical protein
6	6233100	6233579	LOC_Os06g11760	expressed protein
6	6237708	6238080	LOC_Os06g11770	hypothetical protein
6	6240043	6241172	LOC_Os06g11780	MYB family transcription factor, putative, expressed
6	6243660	6247719	LOC_Os06g11790	TLD family protein, putative, expressed
6	6250717	6253708	LOC_Os06g11800	annexin, putative, expressed
6	6253188	6254517	LOC_Os06g11812	wound/stress protein, putative, expressed
6	6255755	6256752	LOC_Os06g11820	expressed protein
6	6277593	6281865	LOC_Os06g11840	trehalose phosphatase, putative, expressed
6	6295652	6299080	LOC_Os06g11850	expressed protein
6	6304588	6306661	LOC_Os06g11860	ethylene-responsive transcription factor, putative, expressed
6	6309925	6310281	LOC_Os06g11870	hypothetical protein
6	6333934	6334978	LOC_Os06g11900	expressed protein
6	6344013	6347676	LOC_Os06g11910	expressed protein

6	6349120	6352936	LOC_Os06g11920	endonuclease/exonuclease/phosphatase family domain containing protein, expressed
6	6355145	6355981	LOC_Os06g11930	SNARE domain containing protein, putative, expressed
6	6358046	6358855	LOC_Os06g11940	dehydration-responsive element binding protein, putative, expressed
6	6369182	6370694	LOC_Os06g11960	2,3-bisphosphoglycerate-dependent phosphoglycerate mutase, putative,
6	6372353	6376374	LOC_Os06g11970	OsMADS63 - MADS-box family gene with MIKC* type-box, expressed
6	6395388	6397221	LOC_Os06g11980	DUF581 domain containing protein, expressed
6	6401419	6407785	LOC_Os06g11990	expressed protein
6	6419602	6420378	LOC_Os06g12000	expressed protein
6	6424294	6424808	LOC_Os06g12010	hypothetical protein
6	6435824	6439822	LOC_Os06g12020	membrane-anchored ubiquitin-fold protein, putative, expressed
6	6440428	6447606	LOC_Os06g12030	glutaredoxin domain containing protein, putative, expressed
6	6452737	6454454	LOC_Os06g12040	mTERF family protein, expressed
6	6458283	6459567	LOC_Os06g12050	mTERF family protein, expressed
6	6461154	6465270	LOC_Os06g12060	mTERF family protein, expressed
6	6465597	6468062	LOC_Os06g12070	mTERF family protein, expressed
6	6472151	6473692	LOC_Os06g12080	mTERF family protein, expressed
6	6475161	6479073	LOC_Os06g12090	miro, putative, expressed
6	6479283	6480980	LOC_Os06g12100	mTERF family protein, expressed
6	6482366	6485677	LOC_Os06g12110	mTERF family protein, expressed
6	6484043	6488553	LOC_Os06g12120	BRASSINOSTEROID INSENSITIVE 1-associated receptor kinase 1 precursor, putative, expressed
6	6489414	6491711	LOC_Os06g12129	expressed protein
6	6492884	6494714	LOC_Os06g12140	expressed protein
6	6495000	6497910	LOC_Os06g12150	shikimate kinase, putative, expressed
6	6503735	6514399	LOC_Os06g12160	AAA-type ATPase family protein, putative, expressed
6	6515548	6519199	LOC_Os06g12170	expressed protein
6	6521987	6523338	LOC_Os06g12180	jacalin-like lectin domain containing protein, expressed
6	6534746	6535897	LOC_Os06g12190	glutaredoxin, putative, expressed
6	6537949	6538677	LOC_Os06g12200	hypothetical protein

6	6556802	6557319	LOC_Os06g12210	helix-loop-helix DNA-binding domain containing protein, expressed
6	6559753	6563394	LOC_Os06g12220	HVA22, putative, expressed
6	6570778	6572727	LOC_Os06g12230	TCP-domain protein, putative, expressed
6	6584405	6585965	LOC_Os06g12240	hypothetical protein
6	6589792	6591799	LOC_Os06g12250	sphingolipid C4-hydroxylase SUR2, putative, expressed
6	6595940	6602476	LOC_Os06g12260	N-rich protein, putative, expressed
6	6603975	6604635	LOC_Os06g12270	hypothetical protein
6	6605479	6608454	LOC_Os06g12280	glycosyl transferase 8 domain containing protein, putative, expressed
6	6612661	6613440	LOC_Os06g12290	glutathione S-transferase GSTU6, putative, expressed
6	6643235	6643552	LOC_Os06g12300	expressed protein
6	6648387	6653005	LOC_Os06g12310	aquaporin protein, putative, expressed
6	6678245	6680864	LOC_Os06g12320	transmembrane amino acid transporter protein, putative, expressed
6	6682411	6685947	LOC_Os06g12330	amino acid transporter, putative, expressed
6	6686569	6687306	LOC_Os06g12340	expressed protein
6	6691061	6695070	LOC_Os06g12350	amino acid transporter, putative, expressed
6	6696666	6700782	LOC_Os06g12360	pentatricopeptide, putative, expressed
6	6701247	6704416	LOC_Os06g12370	OsFtsH6 FtsH protease, homologue of AtFtsH6, expressed
6	6712478	6713221	LOC_Os06g12380	expressed protein
6	6715886	6722017	LOC_Os06g12390	galactosyltransferase family protein, putative, expressed
6	6725306	6734446	LOC_Os06g12400	Homeobox domain containing protein, expressed
6	6736053	6737803	LOC_Os06g12410	GDSL-like lipase/acylhydrolase, putative, expressed
6	6741342	6741945	LOC_Os06g12430	expressed protein
6	6743783	6744849	LOC_Os06g12440	LTPL60 - Protease inhibitor/seed storage/LTP family protein precursor, putative, expressed
6	6748358	6753338	LOC_Os06g12450	soluble starch synthase 2-3, chloroplast precursor, putative, expressed
6	6754294	6755541	LOC_Os06g12455	expressed protein
6	6758181	6762995	LOC_Os06g12460	CSLA3 - cellulose synthase-like family A; mannan synthase, expressed
6	6782326	6783880	LOC_Os06g12490	expressed protein
6	6788986	6790050	LOC_Os06g12500	membrane associated DUF588 domain containing protein, putative,
6	6790516	6792069	LOC_Os06g12510	pentatricopeptide, putative, expressed

6	6793120	6793387	LOC_Os06g12520	hypothetical protein
6	6794150	6796570	LOC_Os06g12530	CS domain containing protein, putative, expressed
6	6796598	6798469	LOC_Os06g12539	expressed protein
6	6801945	6802405	LOC_Os06g12550	expressed protein
6	6809574	6811963	LOC_Os06g12560	zinc finger, C3HC4 type, domain containing protein, expressed
6	6814708	6815190	LOC_Os06g12570	expressed protein
6	6816402	6819861	LOC_Os06g12580	pro-resilin precursor, putative, expressed
6	6822979	6832750	LOC_Os06g12590	protein kinase, putative, expressed
6	6842811	6848564	LOC_Os06g12600	kinase, pfkB family, putative, expressed
6	6866393	6869521	LOC_Os06g12610	auxin efflux carrier component, putative, expressed
6	6878753	6879729	LOC_Os06g12620	expressed protein
6	6892489	6895644	LOC_Os06g12630	glutathione S-transferase, N-terminal domain containing protein,
6	6905844	6908028	LOC_Os06g12649	expressed protein
6	6915783	6916582	LOC_Os06g12660	NHL repeat-containing protein, putative, expressed
6	6923215	6924997	LOC_Os06g12680	RING-H2 finger protein, putative, expressed
6	6928341	6932490	LOC_Os06g12690	DCN1-like protein 2, putative, expressed
6	6937045	6937368	LOC_Os06g12700	conserved hypothetical protein
6	6950487	6950999	LOC_Os06g12730	expressed protein
6	6953695	6956088	LOC_Os06g12740	expressed protein
6	6963552	6963960	LOC_Os06g12760	hypothetical protein
6	6980031	6986313	LOC_Os06g12780	OsDegp10 - Putative Deg protease homologue, expressed
6	6990939	6994556	LOC_Os06g12790	ras-related protein, putative, expressed
6	6995343	6996671	LOC_Os06g12800	expressed protein
6	7000436	7004404	LOC_Os06g12810	proteins of unknown function domain containing protein, expressed
6	7005872	7008652	LOC_Os06g12820	proteins of unknown function domain containing protein, expressed
6	7019819	7022061	LOC_Os06g12830	expressed protein
6	7025603	7027002	LOC_Os06g12840	expressed protein
6	7031413	7032804	LOC_Os06g12850	expressed protein
6	7037048	7038406	LOC_Os06g12860	proteins of unknown function domain containing protein, putative,
6	7043611	7045679	LOC_Os06g12870	leaf senescence related protein, putative, expressed

6	7047258	7052772	LOC_Os06g12876	expressed protein
6	7053689	7056223	LOC_Os06g12882	S phase cyclin A-associated protein in the ER, putative, expressed
6	7059669	7060522	LOC_Os06g12890	expressed protein
6	7067117	7069522	LOC_Os06g12910	XPA-binding protein 2, putative, expressed
6	7072753	7073191	LOC_Os06g12920	hypothetical protein
6	7076782	7078024	LOC_Os06g12930	expressed protein
6	7079677	7082435	LOC_Os06g12940	expressed protein
6	7085090	7088528	LOC_Os06g12950	expressed protein
6	7093925	7096207	LOC_Os06g12960	expressed protein
6	7102791	7106821	LOC_Os06g12980	cyclin, putative, expressed
6	7118829	7120448	LOC_Os06g12990	tRNA methyltransferase, putative, expressed
6	7121120	7123026	LOC_Os06g13000	ankyrin repeat domain-containing protein, chloroplast precursor,
6	7128352	7133515	LOC_Os06g13020	DNA polymerase lambda, putative, expressed
6	7133789	7140003	LOC_Os06g13030	OsLIM - LIM domain protein, putative actin-binding protein and transcription factor, expressed
6	7144204	7146074	LOC_Os06g13040	glycosyl hydrolases family 16, putative, expressed
6	7148961	7158822	LOC_Os06g13050	peroxidase family protein, expressed
6	7159988	7164156	LOC_Os06g13060	heat shock protein DnaJ, putative, expressed
6	7169830	7172482	LOC_Os06g13070	hhH-GPD superfamily base excision DNA repair protein, putative,
6	7181648	7183586	LOC_Os06g13080	spotted leaf 11, putative, expressed
6	7186601	7188076	LOC_Os06g13090	armadillo/beta-catenin repeat family protein, putative, expressed
6	7188393	7191451	LOC_Os06g13100	CREG1 precursor, putative, expressed
6	7201591	7203994	LOC_Os06g13120	expressed protein
6	7205227	7205817	LOC_Os06g13130	expressed protein
6	7209516	7212490	LOC_Os06g13140	WD domain, G-beta repeat domain containing protein, expressed
6	7216473	7217001	LOC_Os06g13150	hypothetical protein
6	7218853	7220283	LOC_Os06g13160	viral-response family protein, expressed
6	7221749	7224410	LOC_Os06g13170	expressed protein
6	7224942	7226417	LOC_Os06g13180	metalloendoproteinase 1 precursor, putative, expressed
6	7230154	7230942	LOC_Os06g13190	expressed protein

6	7231572	7235608	LOC_Os06g13200	proton-dependent oligopeptide transport, putative, expressed
6	7247729	7251058	LOC_Os06g13210	peptide transporter PTR2, putative, expressed
6	7256821	7259221	LOC_Os06g13215	growth regulator related protein, putative, expressed
6	7260407	7264016	LOC_Os06g13220	expressed protein
6	7267160	7269232	LOC_Os06g13230	expressed protein
6	7278365	7282975	LOC_Os06g13240	BURP domain containing protein, expressed
6	7293011	7296554	LOC_Os06g13250	hypothetical protein
6	7306306	7307547	LOC_Os06g13280	O-methyltransferase, putative, expressed
6	7318599	7323201	LOC_Os06g13310	SAM dependent carboxyl methyltransferase, putative, expressed
6	7325614	7327449	LOC_Os06g13320	protein kinase, putative, expressed
6	7333278	7335741	LOC_Os06g13330	expressed protein
6	7338076	7339727	LOC_Os06g13340	expressed protein
6	7341809	7344913	LOC_Os06g13350	SAM dependent carboxyl methyltransferase, putative, expressed
6	7367791	7368195	LOC_Os06g13380	expressed protein
6	7369137	7371782	LOC_Os06g13390	SAM dependent carboxyl methyltransferase, putative, expressed
6	7376286	7376932	LOC_Os06g13410	OsSCP33 - Putative Serine Carboxypeptidase homologue, expressed
6	7378698	7380890	LOC_Os06g13420	OsSCP34 - Putative Serine Carboxypeptidase homologue, expressed
6	7397012	7397462	LOC_Os06g13440	expressed protein
6	7401127	7404267	LOC_Os06g13450	cystathionine beta-lyase, putative, expressed
6	7406037	7408609	LOC_Os06g13460	SAM dependent carboxyl methyltransferase family protein, putative,
6	7417633	7422147	LOC_Os06g13470	SAM dependent carboxyl methyltransferase, putative, expressed
6	7423689	7424045	LOC_Os06g13480	expressed protein
6	7429730	7433791	LOC_Os06g13490	SAM dependent carboxyl methyltransferase domain containing protein,
6	7455885	7456584	LOC_Os06g13510	SAM dependent carboxyl methyltransferase family protein, putative
6	7459467	7461720	LOC_Os06g13520	SAM dependent carboxyl methyltransferase domain containing protein,
6	7468750	7469413	LOC_Os06g13530	benzoate carboxyl methyltransferase, putative, expressed
6	7474908	7475598	LOC_Os06g13550	SAM dependent carboxyl methyltransferase family protein, putative,
6	7480067	7482194	LOC_Os06g13560	SAM dependent carboxyl methyltransferase, putative, expressed
6	7488066	7496669	LOC_Os06g13570	expressed protein
6	7498331	7500517	LOC_Os06g13580	expressed protein

6	7519619	7520480	LOC_Os06g13590	PE-PGRS family protein, putative, expressed
6	7530834	7534857	LOC_Os06g13600	HEAT repeat family protein, putative, expressed
6	7540774	7541864	LOC_Os06g13610	expressed protein
6	7544706	7545167	LOC_Os06g13620	expressed protein
6	7555463	7558367	LOC_Os06g13640	expressed protein
6	7564755	7571033	LOC_Os06g13650	alpha-mannosidase 2, putative, expressed
6	7574978	7581553	LOC_Os06g13660	alanyl-tRNA synthetase, putative, expressed
6	7584780	7590605	LOC_Os06g13670	E2F family transcription factor protein, putative, expressed
6	7591912	7593109	LOC_Os06g13680	B12D protein, putative, expressed
6	7595183	7595952	LOC_Os06g13690	expressed protein
6	7596393	7597214	LOC_Os06g13700	hypothetical protein
6	7600956	7601522	LOC_Os06g13710	glycosyltransferase, putative, expressed
6	7602364	7605812	LOC_Os06g13720	dehydrogenase E1 component domain containing protein, expressed
6	7607108	7607939	LOC_Os06g13730	glutamate receptor precursor, putative, expressed
6	7619834	7623919	LOC_Os06g13750	expressed protein
6	7629547	7631705	LOC_Os06g13760	glycosyl transferase 8 domain containing protein, putative, expressed
6	7642070	7644455	LOC_Os06g13780	expressed protein
6	7647852	7648414	LOC_Os06g13800	expressed protein
6	7648922	7653701	LOC_Os06g13810	pyrophosphate--fructose 6-phosphate 1-phosphotransferase subunit beta, putative, expressed
6	7661691	7670034	LOC_Os06g13820	dynamin, putative, expressed

Supplementary Table S4 List of predicted candidate genes in 1-Mb intervals of the qGE6.2.1 and qGE6.2.2 regions.

qGE6.2.1

Chr	Start	Stop	Gene Name	Putative Function
6	12931779	12934695	LOC_Os06g22290	legume lectins beta domain containing protein, expressed
6	12962817	12966525	LOC_Os06g22330	expressed protein
6	12967302	12969111	LOC_Os06g22340	cytochrome P450, putative, expressed
6	12973921	12978456	LOC_Os06g22350	expressed protein
6	12983166	12983888	LOC_Os06g22360	hypothetical protein
6	12988184	12990508	LOC_Os06g22370	expressed protein
6	12990974	13000940	LOC_Os06g22380	expressed protein
6	13003028	13003793	LOC_Os06g22390	expressed protein
6	13009938	13010619	LOC_Os06g22394	expressed protein
6	13019641	13021760	LOC_Os06g22400	expressed protein
6	13029814	13030182	LOC_Os06g22410	expressed protein
6	13031173	13032066	LOC_Os06g22420	ZOS6-04 - C2H2 zinc finger protein, expressed
6	13041900	13044183	LOC_Os06g22440	SAM dependent carboxyl methyltransferase, putative, expressed
6	13052769	13053171	LOC_Os06g22450	expressed protein
6	13055162	13058027	LOC_Os06g22460	disease resistance protein RPM1, putative, expressed
6	13061315	13062206	LOC_Os06g22470	hypothetical protein
6	13070021	13070935	LOC_Os06g22490	sphingolipid C4-hydroxylase SUR2, putative, expressed
6	13094272	13095005	LOC_Os06g22530	hypothetical protein
6	13097662	13098133	LOC_Os06g22540	expressed protein
6	13101544	13112981	LOC_Os06g22550	transducin family protein, putative, expressed
6	13117696	13120470	LOC_Os06g22560	formyl transferase, putative, expressed
6	13143806	13146070	LOC_Os06g22600	aluminum-activated malate transporter, putative, expressed
6	13148277	13148889	LOC_Os06g22610	heat shock factor protein HSF8, putative, expressed
6	13152632	13154262	LOC_Os06g22620	hypothetical protein
6	13161181	13161417	LOC_Os06g22640	expressed protein
6	13162901	13171017	LOC_Os06g22650	VHS and GAT domain containing protein, expressed

6	13172164	13175101	LOC_Os06g22654	expressed protein
6	13177537	13181742	LOC_Os06g22660	EMB1303, putative, expressed
6	13182291	13189126	LOC_Os06g22670	tesmin/TSO1-like CXC domain containing protein, expressed
6	13193616	13194239	LOC_Os06g22680	hypothetical protein
6	13196296	13198641	LOC_Os06g22690	bundle sheath defective protein 2, putative, expressed
6	13200907	13202281	LOC_Os06g22700	zinc finger family protein, putative, expressed
6	13244910	13245626	LOC_Os06g22760	OsMADS71 - MADS-box family gene with M-alpha type-box, expressed
6	13278774	13279302	LOC_Os06g22800	hypothetical protein
6	13297087	13301279	LOC_Os06g22810	TKL_IRAK_CrRLK1L-1.14 - The CrRLK1L-1 subfamily has homology to the CrRLK1L homolog, expressed
6	13302546	13306574	LOC_Os06g22820	cyclin-dependent kinase F-1, putative, expressed
6	13316042	13318310	LOC_Os06g22829	expressed protein
6	13327566	13332005	LOC_Os06g22850	microfibrillar-associated protein 1, putative, expressed
6	13335912	13337355	LOC_Os06g22860	expressed protein
6	13345333	13349182	LOC_Os06g22870	OsIAA21 - Auxin-responsive Aux/IAA gene family member, expressed
6	13362903	13363249	LOC_Os06g22880	DEFL8 - Defensin and Defensin-like DEFL family
6	13375503	13390470	LOC_Os06g22919	DEFL9 - Defensin and Defensin-like DEFL family, expressed
6	13379395	13379731	LOC_Os06g22925	expressed protein
6	13391513	13393025	LOC_Os06g22940	hypothetical protein
6	13394969	13397303	LOC_Os06g22950	hypothetical protein
6	13407319	13408859	LOC_Os06g22960	aquaporin protein, putative, expressed
6	13411095	13414131	LOC_Os06g22970	expressed protein
6	13414641	13418309	LOC_Os06g22980	CSLD5 - cellulose synthase-like family D, expressed
6	13428642	13431616	LOC_Os06g23010	conserved hypothetical protein
6	13434831	13435939	LOC_Os06g23020	expressed protein
6	13439148	13439922	LOC_Os06g23030	ZF-HD protein dimerisation region containing protein, expressed
6	13440852	13442506	LOC_Os06g23040	expressed protein
6	13444113	13444616	LOC_Os06g23050	expressed protein
6	13447099	13447755	LOC_Os06g23060	DEFL11 - Defensin and Defensin-like DEFL family
6	13472782	13473249	LOC_Os06g23100	expressed protein

6	13476487	13485209	LOC_Os06g23114	copper methylamine oxidase precursor, putative, expressed
6	13498672	13501214	LOC_Os06g23140	copper methylamine oxidase precursor, putative, expressed
6	13510344	13510755	LOC_Os06g23150	hypothetical protein
6	13514667	13521729	LOC_Os06g23160	bacterial transferase hexapeptide domain containing protein, expressed
6	13538870	13540416	LOC_Os06g23180	expressed protein
6	13541654	13544834	LOC_Os06g23190	receptor-like protein kinase 5 precursor, putative, expressed
6	13554710	13555313	LOC_Os06g23210	ribosomal protein L30 containing protein, expressed
6	13557094	13559144	LOC_Os06g23220	GRF zinc finger family protein, expressed
6	13584733	13591992	LOC_Os06g23274	zinc finger, C3HC4 type, domain containing protein, expressed
6	13601951	13604917	LOC_Os06g23290	phosphatidylinositol 3- and 4-kinase family protein, putative, expressed
6	13610067	13610641	LOC_Os06g23300	expressed protein
6	13616319	13616866	LOC_Os06g23310	expressed protein
6	13622966	13623430	LOC_Os06g23330	expressed protein
6	13630101	13631415	LOC_Os06g23350	late embryogenesis abundant protein D-34, putative, expressed
6	13631703	13632940	LOC_Os06g23360	LTPL70 - Protease inhibitor/seed storage/LTP family protein precursor, expressed
6	13635895	13640070	LOC_Os06g23370	expressed protein
6	13647634	13648352	LOC_Os06g23380	IQ calmodulin-binding motif family protein, putative, expressed
6	13649910	13652229	LOC_Os06g23390	IQ calmodulin-binding motif family protein, putative, expressed
6	13669056	13671846	LOC_Os06g23410	expressed protein
6	13674054	13676418	LOC_Os06g23420	exostosin family domain containing protein, expressed
6	13677506	13681875	LOC_Os06g23430	signal recognition particle 19 kDa protein, putative, expressed
6	13685422	13686180	LOC_Os06g23440	eukaryotic translation initiation factor 1A, putative, expressed
6	13690553	13697926	LOC_Os06g23460	KH domain containing protein, putative, expressed
6	13699712	13700325	LOC_Os06g23470	hypothetical protein
6	13701072	13701446	LOC_Os06g23480	expressed protein
6	13704784	13722973	LOC_Os06g23504	DTW domain containing protein, putative, expressed
6	13738447	13739456	LOC_Os06g23540	expressed protein
6	13743329	13745442	LOC_Os06g23560	anthocyanidin 5,3-O-glucosyltransferase, putative, expressed
6	13750549	13751946	LOC_Os06g23570	expressed protein
6	13755124	13755576	LOC_Os06g23590	expressed protein

6	13762013	13767164	LOC_Os06g23600	expressed protein
6	13775352	13775953	LOC_Os06g23620	expressed protein
6	13786384	13788325	LOC_Os06g23640	expressed protein
6	13804017	13805815	LOC_Os06g23650	no apical meristem protein, putative, expressed
6	13835593	13839057	LOC_Os06g23684	tyrosine aminotransferase, putative, expressed
6	13871663	13873423	LOC_Os06g23740	expressed protein
6	13877622	13878188	LOC_Os06g23750	expressed protein
6	13883250	13884184	LOC_Os06g23760	acyl-CoA oxidase, putative, expressed
6	13885410	13888877	LOC_Os06g23770	expressed protein
6	13888924	13889646	LOC_Os06g23775	expressed protein
6	13892471	13897688	LOC_Os06g23780	glutaryl-CoA dehydrogenase, mitochondrial precursor, putative, expressed
6	13900584	13901962	LOC_Os06g23790	expressed protein
6	13905082	13909018	LOC_Os06g23800	flavin-containing monooxygenase family protein, putative, expressed
6	13915101	13915751	LOC_Os06g23810	expressed protein
6	13937872	13938395	LOC_Os06g23850	expressed protein
6	13949417	13951281	LOC_Os06g23870	acyl-CoA dehydrogenase domain protein, putative, expressed
6	13956961	13959295	LOC_Os06g23880	hypothetical protein

qGE6.2.2

Chr	Start	Stop	Gene Name	Putative Function
6	16052471	16056775	LOC_Os06g28250	expressed protein
6	16058398	16060555	LOC_Os06g28260	expressed protein
6	16070798	16072705	LOC_Os06g28300	zinc knuckle family protein, expressed
6	16168793	16169592	LOC_Os06g28420	expressed protein
6	16195179	16195513	LOC_Os06g28470	expressed protein
6	16198162	16200384	LOC_Os06g28480	polygalacturonase inhibitor 1 precursor, putative, expressed
6	16217263	16219073	LOC_Os06g28520	expressed protein
6	16224024	16225310	LOC_Os06g28524	expressed protein
6	16245674	16247171	LOC_Os06g28550	nmrA-like family domain containing protein, expressed
6	16251104	16252882	LOC_Os06g28560	expressed protein

6	16264254	16270836	LOC_Os06g28590	bg55, putative, expressed
6	16276332	16278036	LOC_Os06g28600	expressed protein
6	16302034	16305078	LOC_Os06g28630	expressed protein
6	16329395	16331464	LOC_Os06g28670	polygalacturonase, putative, expressed
6	16335038	16335905	LOC_Os06g28680	expressed protein
6	16339515	16340768	LOC_Os06g28690	expressed protein
6	16352388	16353257	LOC_Os06g28720	expressed protein
6	16353727	16354521	LOC_Os06g28730	expressed protein
6	16363079	16366463	LOC_Os06g28740	expressed protein
6	16381270	16382990	LOC_Os06g28760	hypothetical protein
6	16385565	16387434	LOC_Os06g28770	expressed protein
6	16400699	16432410	LOC_Os06g28820	cycloartenol synthase, putative, expressed
6	16440845	16444414	LOC_Os06g28840	expressed protein
6	16481194	16485465	LOC_Os06g28890	expressed protein
6	16502251	16503405	LOC_Os06g28940	hypothetical protein
6	16504514	16505369	LOC_Os06g28950	expressed protein
6	16506995	16507947	LOC_Os06g28960	expressed protein
6	16509328	16512902	LOC_Os06g28970	bolA, putative, expressed
6	16534747	16535159	LOC_Os06g29010	expressed protein
6	16579007	16583086	LOC_Os06g29080	expressed protein
6	16588693	16590109	LOC_Os06g29100	expressed protein
6	16593797	16599300	LOC_Os06g29110	MLO domain containing protein, putative, expressed
6	16604790	16616264	LOC_Os06g29120	STE_PAK_Ste20_STLK.5 - STE kinases include homologs to sterile 7, sterile 11 and sterile 20 from yeast, expressed
6	16649544	16653296	LOC_Os06g29180	erythronate-4-phosphate dehydrogenase domain containing protein, expressed
6	16658542	16658778	LOC_Os06g29190	expressed protein
6	16676478	16683139	LOC_Os06g29220	erythronate-4-phosphate dehydrogenase domain containing protein, expressed
6	16687151	16687942	LOC_Os06g29240	expressed protein
6	16693757	16694669	LOC_Os06g29250	expressed protein
6	16719384	16719848	LOC_Os06g29290	pentatricopeptide, putative, expressed

6	16732924	16745662	LOC_Os06g29310	ulp1 protease family protein, putative, expressed
6	16774133	16785410	LOC_Os06g29350	myosin, putative, expressed
6	16785962	16787113	LOC_Os06g29360	expressed protein
6	16791220	16793141	LOC_Os06g29370	expressed protein
6	16793634	16799776	LOC_Os06g29380	phospholipid-transporting ATPase, putative, expressed
6	16810191	16810436	LOC_Os06g29390	expressed protein
6	16825601	16836210	LOC_Os06g29400	surp module family protein, putative, expressed
6	16852004	16858873	LOC_Os06g29430	cytidine and deoxycytidylate deaminase zinc-binding region family protein, expressed
6	16862021	16864413	LOC_Os06g29440	expressed protein
6	16877329	16878690	LOC_Os06g29450	expressed protein
6	16879347	16881668	LOC_Os06g29460	expressed protein
6	16896515	16897661	LOC_Os06g29470	peroxidase precursor, putative, expressed
6	16910985	16916624	LOC_Os06g29490	expressed protein
6	16927219	16928806	LOC_Os06g29540	expressed protein
6	16933182	16936296	LOC_Os06g29560	expressed protein
6	16960716	16961852	LOC_Os06g29590	expressed protein
6	16983848	16984156	LOC_Os06g29620	hypothetical protein
6	17003628	17010230	LOC_Os06g29650	CDP-diacylglycerol--inositol 3-phosphatidyltransferase 1, putative, expressed
6	17020710	17021075	LOC_Os06g29670	expressed protein
6	17029405	17030321	LOC_Os06g29679	expressed protein
6	17035080	17036915	LOC_Os06g29690	dynein light chain type 1 domain containing protein, expressed
6	17044170	17046351	LOC_Os06g29700	OsFBD11 - F-box and FBD domain containing protein, expressed
6	17054545	17066625	LOC_Os06g29710	OsFBD12 - F-box and FBD domain containing protein, expressed
6	17066919	17068618	LOC_Os06g29730	RALFL28 - Rapid ALkalinization Factor RALF family protein precursor, expressed
6	17077761	17080192	LOC_Os06g29740	OsFBX197 - F-box domain containing protein, expressed

Supplementary Table S5 List of predicted candidate genes in 1-Mb intervals of the qGE4.1 region.

Chr	Start	Stop	Gene Name	Description
4	19651659	19656024	LOC_Os04g32620	ethylene-responsive transcription factor ERF114, putative, expressed
4	19669788	19670995	LOC_Os04g32640	hypothetical protein
4	19677039	19682186	LOC_Os04g32650	tRNA synthetase class II core domain containing protein, expressed
4	19684934	19688086	LOC_Os04g32660	cytochrome b-c1 complex subunit Rieske, mitochondrial precursor, putative, expressed
4	19690552	19694678	LOC_Os04g32670	exostosin family domain containing protein, expressed
4	19693409	19694678	LOC_Os04g32680	POEI20 - Pollen Ole e I allergen and extensin family protein precursor,
4	19695368	19709974	LOC_Os04g32690	expressed protein
4	19710889	19711503	LOC_Os04g32700	LEML3 - Anther-specific LEM1 family protein precursor, expressed
4	19712823	19714034	LOC_Os04g32710	40S ribosomal protein S27, putative, expressed
4	19735871	19740198	LOC_Os04g32740	hydrolase, NUDIX family, domain containing protein, expressed
4	19773223	19773949	LOC_Os04g32780	hypothetical protein
4	19790424	19791835	LOC_Os04g32790	AP2 domain containing protein, expressed
4	19796270	19798423	LOC_Os04g32800	basic proline-rich protein, putative, expressed
4	19812328	19814330	LOC_Os04g32810	expressed protein
4	19819398	19820459	LOC_Os04g32820	expressed protein
4	19822056	19822949	LOC_Os04g32830	expressed protein
4	19826312	19827902	LOC_Os04g32840	formin-like protein 20, putative, expressed
4	19835131	19836871	LOC_Os04g32850	basic proline-rich protein, putative, expressed
4	19841565	19844276	LOC_Os04g32860	expressed protein
4	19849118	19850578	LOC_Os04g32870	pentatricopeptide repeat-containing protein, putative, expressed
4	19850971	19859052	LOC_Os04g32880	CBS domain containing membrane protein, putative, expressed
4	19886676	19892492	LOC_Os04g32920	potassium transporter, putative, expressed
4	19893384	19896711	LOC_Os04g32930	DNA cross-link repair 1A protein, putative, expressed
4	19899895	19903657	LOC_Os04g32940	Leucine Rich Repeat family protein, expressed
4	19913228	19916749	LOC_Os04g32950	calreticulin precursor protein, putative, expressed
4	19920789	19929902	LOC_Os04g32960	TUDOR protein with multiple SNc domains, putative, expressed

4	19933108	19937566	LOC_Os04g32970	OTU-like cysteine protease family protein, putative, expressed
4	19940102	19942156	LOC_Os04g32980	protein binding protein, putative, expressed
4	19984084	19984644	LOC_Os04g33020	expressed protein
4	19994476	19996281	LOC_Os04g33030	protein binding protein, putative, expressed
4	20006128	20009276	LOC_Os04g33040	alpha-amylase precursor, putative, expressed
4	20009913	20015529	LOC_Os04g33050	HIRAN domain containing protein, putative, expressed
4	20015750	20019634	LOC_Os04g33060	NAD dependent epimerase/dehydratase family protein, putative,
4	20020086	20020355	LOC_Os04g33070	expressed protein
4	20021233	20025228	LOC_Os04g33080	protein phosphatase 2C, putative, expressed
4	20038759	20039182	LOC_Os04g33100	hypothetical protein
4	20045882	20046918	LOC_Os04g33110	expressed protein
4	20047507	20048091	LOC_Os04g33115	expressed protein
4	20057589	20058305	LOC_Os04g33130	expressed protein
4	20061940	20064227	LOC_Os04g33140	expressed protein
4	20065193	20066677	LOC_Os04g33150	desiccation-related protein PCC13-62 precursor, putative, expressed
4	20067814	20068212	LOC_Os04g33160	hypothetical protein
4	20072917	20073300	LOC_Os04g33170	hypothetical protein
4	20074215	20074457	LOC_Os04g33180	expressed protein
4	20077759	20083076	LOC_Os04g33190	AMP-binding enzyme, putative, expressed
4	20083916	20086861	LOC_Os04g33200	heparan-alpha-glucosaminide N-acetyltransferase, putative, expressed
4	20094182	20099729	LOC_Os04g33210	ERD1 protein, chloroplast precursor, putative, expressed
4	20099031	20103020	LOC_Os04g33220	SAM domain family protein, expressed
4	20105082	20108469	LOC_Os04g33230	hypothetical protein
4	20109574	20110741	LOC_Os04g33240	sex determination protein tasselseed-2, putative, expressed
4	20114964	20115457	LOC_Os04g33250	conserved hypothetical protein
4	20117269	20122227	LOC_Os04g33260	expressed protein
4	20124675	20131224	LOC_Os04g33280	expressed protein
4	20134769	20135921	LOC_Os04g33290	cysteine protease, putative, expressed
4	20138880	20141866	LOC_Os04g33300	amino acid kinase, putative, expressed
4	20144021	20145529	LOC_Os04g33310	expressed protein

4	20158993	20161450	LOC_Os04g33320	expressed protein
4	20169585	20171246	LOC_Os04g33390	prephenate dehydratase domain containing protein, expressed
4	20187930	20189402	LOC_Os04g33330	expressed protein
4	20190337	20190988	LOC_Os04g33340	expressed protein
4	20195358	20196213	LOC_Os04g33350	expressed protein
4	20200072	20201885	LOC_Os04g33360	gibberellin 2-beta-dioxygenase 7, putative, expressed
4	20211340	20213320	LOC_Os04g33370	cytochrome P450, putative, expressed
4	20214939	20215652	LOC_Os04g33380	expressed protein
4	20221253	20223074	LOC_Os04g33450	expressed protein
4	20224555	20226415	LOC_Os04g33440	DNA-binding protein S1FA, putative, expressed
4	20226884	20227249	LOC_Os04g33430	expressed protein
4	20229222	20230813	LOC_Os04g33420	DNA-binding protein S1FA, putative, expressed
4	20236216	20238126	LOC_Os04g33400	OsFBLD2 - F-box, LRR and FBD domain containing protein, expressed
4	20239790	20243460	LOC_Os04g33460	1,4-alpha-glucan-branching enzyme 2, chloroplast precursor, putative,
4	20245217	20249961	LOC_Os04g33470	Ser/Thr protein phosphatase family protein, putative, expressed
4	20250089	20255825	LOC_Os04g33480	histone deacetylase, putative, expressed

Supplementary Table S6 Plants with high grain elongation ratio and low grain elongation ratio.

The letters A and a represent Basmati's and PTT1's marker alleles, respectively.

No.	SSIIa	SBEIII	SBEIIa	GE ratio
118	Aa	AA	Aa	2.37
5	Aa	Aa	aa	2.30
89	Aa	Aa	Aa	2.29
95	AA	Aa	AA	2.29
148	Aa	AA	AA	2.24
73	AA	Aa	Aa	2.15
101	Aa	Aa	aa	2.11
179	AA	Aa	aa	2.08
71	Aa	Aa	aa	2.06
35	AA	Aa	Aa	2.04
126	AA	Aa	AA	2.04
149	Aa	Aa	Aa	2.01
137	AA	AA	Aa	2.00
130	AA	Aa	Aa	2.00
139	Aa	Aa	AA	1.99
52	AA	Aa	Aa	1.98
98	AA	Aa	AA	1.96
8	AA	Aa	AA	1.95
79	Aa	Aa	Aa	1.95
37	Aa	Aa	aa	1.93
18	Aa	Aa	Aa	1.64
196	Aa	Aa	Aa	1.63
153	Aa	Aa	Aa	1.63
177	aa	aa	Aa	1.62
176	aa	aa	aa	1.62
161	Aa	Aa	Aa	1.62
40	aa	aa	Aa	1.61
83	aa	aa	aa	1.61
19	Aa	aa	aa	1.60
168	aa	aa	aa	1.59
120	Aa	aa	aa	1.57
169	aa	aa	Aa	1.56
22	aa	Aa	aa	1.55
156	aa	aa	aa	1.55
6	aa	aa	AA	1.47
184	Aa	Aa	aa	1.44
100	Aa	aa	aa	1.39
25	aa	aa	aa	1.39
108	aa	aa	Aa	1.39
56	aa	aa	Aa	1.35

Supplementary Table S7 Grain elongation ratios and marker genotypes for Wx, SSI, SSIIa, SBEIII and SBEIIa among 170 F2 plants

Sample Name	Wx	SSI	SSIIa	SBEIII	SBEIIa	Phenotype (GE ratio)
1	B	B	B	B	A	1.87
2	H	H	H	H	H	1.78
4	H	H	H	B	A	1.78
5	H	H	H	H	A	2.30
6	A	A	A	A	B	1.47
7	B	B	B	H	H	1.89
8	B	B	B	H	B	1.95
10	B	B	B	H	H	1.81
11	H	H	H	H	H	1.67
12	B	B	H	H	A	1.96
13	H	H	H	H	B	1.83
14	H	H	H	H	H	1.86
15	B	B	B	B	H	1.69
16	H	B	B	H	A	1.39
17	B	B	H	A	B	1.77
18	H	H	H	H	H	1.83
19	H	H	H	A	A	1.60
21	H	H	H	H	A	1.80
22	A	A	A	H	A	1.55
23	H	B	H	B	B	1.85
25	H	H	A	A	A	1.39
26	H	H	H	B	B	1.63
27	A	A	A	H	H	1.91
28	H	H	H	B	H	1.80
31	H	H	A	A	H	1.66
32	A	A	H	H	A	1.78
33	H	H	B	B	H	1.65
34	A	A	A	A	B	1.99
35	B	B	B	H	H	2.04
36	H	H	H	H	A	1.73
37	H	H	H	H	A	1.93
38	H	H	H	A	A	1.42
39	H	A	A	A	A	1.41
40	B	H	A	A	H	1.61
41	H	H	H	H	H	1.70
42	B	B	B	B	H	1.79
43	A	A	A	A	A	1.76
44	H	A	H	H	H	1.53
45	H	H	H	H	A	1.79
46	B	B	H	H	H	1.82
47	A	A	H	A	H	1.70
48	A	H	H	H	B	1.66
49	A	A	A	A	A	1.90
50	H	H	H	H	H	1.55
51	H	H	H	H	H	1.62
52	H	H	B	H	H	1.98
54	H	B	B	B	H	1.91
55	H	H	H	H	A	1.82
56	A	A	A	A	H	1.35
57	H	B	B	B	A	1.71
59	B	B	H	H	H	1.51
60	A	H	H	H	H	1.86

62	A	A	A	A	A	2.09
63	A	H	H	H	A	1.78
64	H	H	H	H	B	1.65
65	H	B	H	H	H	1.63
66	H	H	H	H	A	1.78
67	H	H	H	H	B	1.88
69	H	H	H	A	A	1.60
70	H	H	H	A	H	1.70
71	A	H	H	H	A	2.06
73	A	H	B	H	H	2.15
74	H	H	A	H	H	1.91
75	H	H	H	B	B	1.87
76	A	A	A	A	A	1.70
78	H	H	B	B	H	1.88
79	H	H	H	H	H	1.95
80	A	A	H	H	H	1.66
82	A	A	A	A	A	1.51
83	H	H	A	A	A	1.61
84	H	H	A	A	A	1.77
85	H	H	B	H	A	1.83
87	H	H	B	B	B	1.86
88	B	B	B	B	H	1.78
89	A	H	H	H	H	2.29
90	H	H	A	A	A	1.61
91	A	H	A	A	H	1.76
92	B	B	H	H	H	1.83
93	A	A	A	A	A	1.74
94	H	A	A	A	H	1.50
95	A	A	B	H	B	2.29
96	H	B	H	H	A	1.63
97	H	H	A	A	A	1.73
98	B	B	B	H	B	1.96
99	A	A	H	H	A	1.86
100	A	A	H	A	A	1.39
101	A	H	H	H	A	2.11
102	A	A	A	H	H	1.72
103	B	B	H	H	B	1.72
104	H	B	H	H	B	1.66
105	H	H	H	H	H	1.74
106	B	H	H	A	A	1.72
108	A	A	A	A	H	1.39
110	H	H	H	H	B	1.74
111	H	H	H	H	H	1.87
113	H	H	H	H	B	1.82
114	H	H	H	H	H	1.83
115	A	A	H	B	H	1.72
117	H	B	B	B	H	1.88
118	B	B	H	B	H	2.37
119	B	B	H	A	B	1.68
120	H	H	H	A	A	1.57
121	H	H	H	H	A	1.77
122	A	A	B	H	A	1.68
124	H	B	H	H	B	1.87
125	A	H	B	B	H	1.65
126	B	B	B	H	B	2.04
127	H	H	H	H	H	1.62
129	A	A	H	H	H	1.66
130	B	B	B	H	H	2.00
131	A	A	A	A	A	1.71
132	H	H	H	H	A	1.82

133	A	H	H	B	A	1.75
134	A	H	H	H	B	1.79
135	A	A	B	H	H	1.65
136	B	B	B	B	A	1.76
137	B	B	B	B	H	2.00
139	H	H	H	H	B	1.99
140	H	H	A	A	H	1.73
141	H	H	H	A	H	1.82
142	B	B	B	H	H	1.90
143	H	H	H	H	H	1.61
144	B	H	H	H	H	1.83
145	H	H	A	A	B	1.49
146	A	A	A	A	H	1.90
147	B	B	B	B	H	1.66
148	A	H	H	B	B	2.24
149	H	H	H	H	H	2.01
150	B	B	B	B	H	1.92
151	H	H	H	H	H	1.57
152	B	B	B	B	H	1.76
153	B	H	H	H	H	1.63
154	A	A	H	H	H	1.58
155	A	A	A	H	A	1.51
156	A	A	A	A	A	1.55
157	H	H	H	H	H	1.69
158	H	A	H	H	H	1.92
159	H	H	B	B	H	1.45
160	A	B	B	B	H	1.67
161	A	H	H	H	H	1.62
162	H	H	H	A	A	1.73
164	H	H	H	H	H	1.76
167	B	B	B	B	B	1.93
168	A	A	A	A	A	1.59
169	A	A	A	A	H	1.56
170	A	A	A	A	H	1.84
171	B	B	B	B	H	1.84
172	H	H	H	H	A	1.89
173	H	H	H	H	B	1.75
174	A	H	A	A	H	1.73
175	A	H	H	H	H	1.64
176	H	H	A	A	A	1.62
177	A	A	A	A	H	1.62
178	H	H	H	H	A	1.88
179	H	H	B	H	A	2.08
180	B	H	H	H	H	1.94
181	A	A	H	H	A	1.87
183	H	H	H	H	H	1.88
184	A	H	H	H	A	1.44
185	A	H	H	H	A	1.94
186	A	H	H	H	H	1.89
187	B	B	H	H	A	1.89
188	A	A	H	H	A	1.80
189	B	B	B	B	A	1.76
190	B	B	H	H	A	1.65
191	A	A	H	H	H	1.65
193	H	H	H	H	A	1.67
194	H	H	B	H	B	1.85
195	B	H	H	H	H	1.73
196	H	H	H	H	H	1.63
